# A novel circulating miRNA panel for early differential diagnosis of pulmonary tuberculosis from lung cancer with similar radiographic presentations

**DOI:** 10.3389/fmed.2025.1660291

**Published:** 2025-10-08

**Authors:** Xiao Liu, Wentao Wang, Yurong Fu, Zhengjun Yi

**Affiliations:** ^1^School of Laboratory Medicine, Shandong Second Medical University, Weifang, China; ^2^Shandong Advanced Academy Engineering Research Institute for Precision Medicine Innovation and Transformation of Infectious Disease, Weifang, China; ^3^School of Basic Medical Sciences, Shandong Second Medical University, Weifang, China; ^4^Affiliated Hospital of Shandong Second Medical University, Weifang, China

**Keywords:** miRNA, pulmonary tuberculosis, lung cancer, differential diagnosis, biomarker panel

## Abstract

This study aimed to establish a novel diagnostic approach based on a circulating microRNA (miRNA) panel to reliably distinguish pulmonary tuberculosis (PTB) from lung cancer (LC), particularly in patients presenting with overlapping radiographic features. A total of 592 participants were enrolled. In the discovery phase, miRNA expression profiles from 284 individuals, including PTB patients, LC patients, and healthy controls (HC), were analyzed to screen potential biomarkers. Candidate miRNAs were subsequently validated in an independent cohort of 308 plasma samples. The analysis revealed significant upregulation of hsa-miR-342-3p, hsa-miR-199a-3p, and hsa-miR-199b-3p in PTB compared with LC. A diagnostic panel incorporating these three miRNAs demonstrated robust performance, achieving an area under the receiver operating characteristic curve (AUC) of 0.911 (95% confidence interval [CI]: 0.852–0.952) in the training set and 0.886 (95% CI: 0.780–0.953) in the validation set, with a sensitivity of 0.879. This miRNA panel outperformed the World Health Organization -endorsed Xpert MTB/RIF assay, effectively identifying early-stage PTB cases without cavity formation. These findings underscore the potential of miRNA–based diagnostics as a non-invasive and highly accurate tool for differentiating PTB from LC in patients with comparable imaging presentations, addressing a critical gap in pulmonary disease management.

## Highlights

- Existing diagnostic approaches for PTB and lung cancer LC are limited by overlapping radiographic manifestations, reliance on invasive procedures, and suboptimal sensitivity.
- Plasma analysis revealed increased overexpression of hsa-miR-342-3p, hsa-miR-199a-3p, and hsa-miR-199b-3p in patients with PTB compared with those with LC.
- A diagnostic model incorporating these three miRNAs achieved high discriminatory power (training cohort AUC 0.911, validation cohort AUC 0.886), supporting its application for accurate, early, and non-invasive detection of PTB.

## Introduction

Tuberculosis (TB) remains a pressing global health challenge and is still the leading cause of death from a single infectious agent. PTB, the most common clinical form, poses considerable diagnostic difficulties because its radiographic manifestations often overlap with those of LC. This similarity, combined with the limitations of existing non-invasive diagnostic techniques, usually leads to the misdiagnosis of PTB as LC (1–5). Accurate differentiation between PTB and LC, particularly in patients with comparable imaging features, is therefore crucial for timely treatment initiation and improved clinical outcomes.

Conventional non-invasive diagnostic methods, such as sputum smear microscopy and culture, are hampered by low sensitivity and long turnaround times. The Xpert MTB/RIF assay offers greater diagnostic accuracy; however, its high cost restricts its widespread use, especially in low-resource settings (6). Invasive procedures such as tissue biopsy can provide a definitive diagnosis but are not always accessible in resource-limited healthcare systems (7). These limitations underscore the urgent need for a rapid, reliable, and cost-effective non-invasive diagnostic strategy for PTB.

MicroRNAs, a class of small non-coding RNAs approximately 20–25 nucleotides in length, have emerged as highly stable biomarkers detectable in plasma and other body fluids, making them attractive candidates for non-invasive diagnostics (8, 9). Circulating miRNAs have shown diagnostic potential across multiple diseases, including malignancies and infectious conditions (10–12). Specific miRNAs are linked to the molecular pathogenesis of both LC and TB. For instance, hsa-miR-21–5p and hsa-miR-196b-5p have been implicated in the progression of LC, while hsa-miR-495 and hsa-miR-543 contribute to LC invasiveness (13–15). In TB, miRNAs such as hsa-miR-342, hsa-miR-222-3p, hsa-miR-431-3p, and hsa-miR-1303 play essential roles in regulating host innate immune responses (16, 17).

In this study, plasma miRNA profiles from 308 participants were analyzed, and a diagnostic classifier was developed using binary logistic regression to differentiate PTB from LC. The miRNA panel demonstrated higher sensitivity compared with conventional diagnostic methods, including Xpert MTB/RIF, sputum smear microscopy, and culture. These findings highlight the potential of miRNA–based assays as a precise, non-invasive strategy for improving early and accurate differentiation of PTB and LC.

## Methods

### Patient recruitment and sample collection

A total of 308 peripheral blood samples were collected from patients at Weifang No. 2 People’s Hospital between March 2021 and March 2024. Blood was drawn into EDTA-K_2_ anticoagulant tubes and centrifuged at 3000 rpm for 15 min at room temperature. The plasma fraction was then carefully separated and stored for subsequent RNA extraction. In addition to these clinical samples, miRNA expression profiles were obtained from publicly available datasets in the Gene Expression Omnibus (GEO) database, including GSE116542^1^, GSE31568, and GSE61741 (18, 19).

### Reverse transcription-quantitative polymerase chain reaction

Total RNA was isolated from plasma samples using TRIzol™ LS Reagent (Thermo Fisher Scientific, Waltham, MA, United States) in accordance with the manufacturer’s instructions. Complementary DNA (cDNA) was synthesized from the extracted RNA using the PrimeScript™ II 1st Strand cDNA Synthesis Kit (Takara Bio, Shiga, Japan). Quantitative real-time PCR (qRT-PCR) was then conducted under the following thermal cycling conditions: initial denaturation at 95 °C for 30 s, followed by 40 amplification cycles consisting of denaturation at 95 °C for 5 s and annealing/extension at 60 °C for 30 s.

Primers specific for U6 small nuclear RNA (used as the endogenous control) and the three target miRNAs were synthesized by Sangon Biotech (Shanghai, China). Relative expression levels of miRNAs were calculated using the 2^−ΔCt^ method, where ΔCt = Ct (miRNA) – Ct (U6).

The target miRNA sequences, sourced from the miRDB database, were as follows:

- hsa-miR-342-3p (23 bp): 5′-TCTCACACAGAAATCGCACCCGT-3′.
- hsa-miR-199a-3p (22 bp): 5′-ACAGTAGTCTGCACATTGGTTA-3′.
- hsa-miR-199b-3p (22 bp): 5′-ACAGTAGTCTGCACATTGGTTA-3′.

Verification against the GenBank database identified *Mus musculus* U6 small nuclear RNA-RnU6 (GeneID: 19862, 107 bp):

5′-GTGCTCGCTTCGGCAGCACATATACTAAAATTGGAACGATACAGAGAAGATTAGCATGGCCCCTGCGCAAGGATGACACGCAAATTCGTGAAGCGTTCCATATTTTT-3′.

### Identification of candidate miRNA biomarkers

Differentially expressed miRNAs were identified using the GEO2R online analysis tool.^2^ For the LC group, the selection threshold was set at |log₂ fold change (FC)| ≥ 0, whereas for the PTB group, the threshold was |log₂FC| ≥ 1 with a significance level of *p* ≤ 0.05. Here, FC represents the ratio of the mean gene expression level in patients compared with HC. To maximize the inclusion of potentially informative biomarkers, no false discovery rate (FDR) adjustment or other multiple-testing corrections were applied during the initial screening, and statistical significance was defined as *p* ≤ 0.05. Furthermore, candidate miRNAs were required to show opposing expression patterns between the PTB and LC groups to enhance their diagnostic relevance.

### Diagnostic performance and clinical net benefit analysis

A binary logistic regression model was established using SPSS software, version 27.0.1 (IBM Corp., Armonk, NY, USA). The diagnostic performance of the model was assessed by generating receiver operating characteristic (ROC) curves and calculating the AUC with MedCalc Statistical Software, version 20.022 (MedCalc Software bvba, Ostend, Belgium). Similarly, decision curve analysis (DCA) was conducted using R software, version 3.5.0 (R Development Core Team, Vienna, Austria), to evaluate the clinical net benefit of the predictive model.

### Functional enrichment analysis of miRNA target mRNAs

Putative mRNA targets of the selected miRNAs were predicted using three publicly available databases: TargetScan version 7.2^3^, miRWalk^4^, and miRDB.^5^ Functional enrichment analyzes of the predicted target genes were then performed using Gene Ontology (GO) annotations and Kyoto Encyclopedia of Genes and Genomes (KEGG) pathway analysis via the Sangerbox online platform.^6^

### Statistical analysis

An independent *t*-test was applied to evaluate differences between two groups, whereas one-way analysis of variance (ANOVA) was used for comparisons involving more than two groups. Sensitivity comparisons among diagnostic methods were performed according to the following criteria: when the sample size was *n* ≥ 40 and all expected frequencies (T) were ≥ 5, Pearson’s chi-square test was used; when *n* ≥ 40 with at least one expected frequency of 1 ≤ T < 5, the continuity-corrected chi-square test was applied; and when *n* < 40 or any expected frequency was T < 1, Fisher’s exact test was employed. All statistical analyzes and graphical visualizations were conducted using GraphPad Prism software, version 8.0.2 (GraphPad Software, San Diego, CA, United States), and R software, version 3.5.0 (R Development Core Team, Vienna, Austria). A *p*-value of < 0.05 was considered statistically significant.

## Results

### Study participants and characteristics

A total of 308 participants were initially recruited for this study, comprising 128 patients with PTB, 109 patients with LC, and 71 HC. PTB cases were identified based on clinical symptoms and chest imaging findings consistent with tuberculosis. Among these, 86 patients were confirmed by positive results from *Mycobacterium tuberculosis* (MTB) culture, acid-fast bacilli (AFB) smear microscopy, the TB-LAMP assay, or the Xpert MTB/RIF test. The remaining 42 PTB cases were diagnosed based on clinical presentation, chest radiographic features, and a favorable response to anti-TB therapy, in accordance with the Chinese Clinical Guideline for Diagnosis of PTB (WS 288-2017). LC cases were confirmed primarily through histological and/or cytological examination, supplemented by clinical evaluation when necessary. The HC group included individuals without respiratory symptoms and those with normal chest imaging results obtained within 3 months before enrollment. None of the participants had a history of PTB or other malignancies, and all were free from medical treatment within the 4 weeks preceding recruitment.

**Figure 1:**
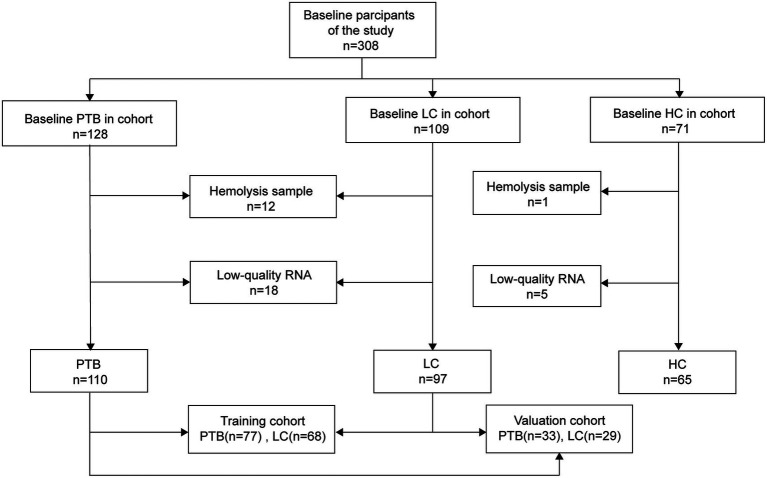
Flow diagram illustrating the sample screening process. PTB, pulmonary tuberculosis; LC, lung cancer; HC, healthy control.

To minimize potential confounding, all participants were screened to exclude co-infections such as HIV and hepatitis B virus. Plasma samples that were hemolyzed or failed to meet RNA quality control criteria were excluded, resulting in 272 eligible samples for downstream analysis. The sample selection process is outlined in Figure 1, and the demographic and clinical characteristics of the 272 included participants are summarized in Table 1.

**Table 1:** Characteristics of the study populations.

Characteristics	Category/Measure	Study population		
		PTB (*n* = 110)	LC (*n* = 97)	HC (*n* = 65)
Age at sampling	Mean±SEM	44.32 ± 1.82	61.18 ± 0.99	41.23 ± 2.12
Gender–counts	Male/Female	80/30	54/43	45/20
Clinical symptoms	Fever	21	3	0
	Fatigue and night sweats	4	1	0
	Cough	78	72	0
	Expectoration	67	66	0
	Haemoptysis	18	11	0
	Chest pain	10	13	0
	Asymptomatic	23	23	65
Presence of cavities	Positive	38	NA	NA
	Negative	72		
AFB smear	Positive	25	NA	NA
	Negative	60		
	NA	25		
TB-LAMP	Positive	39	NA	NA
	Negative	28		
	NA	43		
X-pert	Positive	58	NA	NA
	Negative	37		
	NA	15		
Culture	Positive	57	NA	NA
	Negative	42		
	NA	11		

SEM, standard Error of Measurement; PTB, pulmonary tuberculosis; n, number; LC, lung cancer; HC, healthy control; SEM, Standard Error of the Mean; TB, tuberculosis; NA, not available; AFB, acid-fast bacteria.

### Identification of a 3-miRNA signature

**Figure 2:**
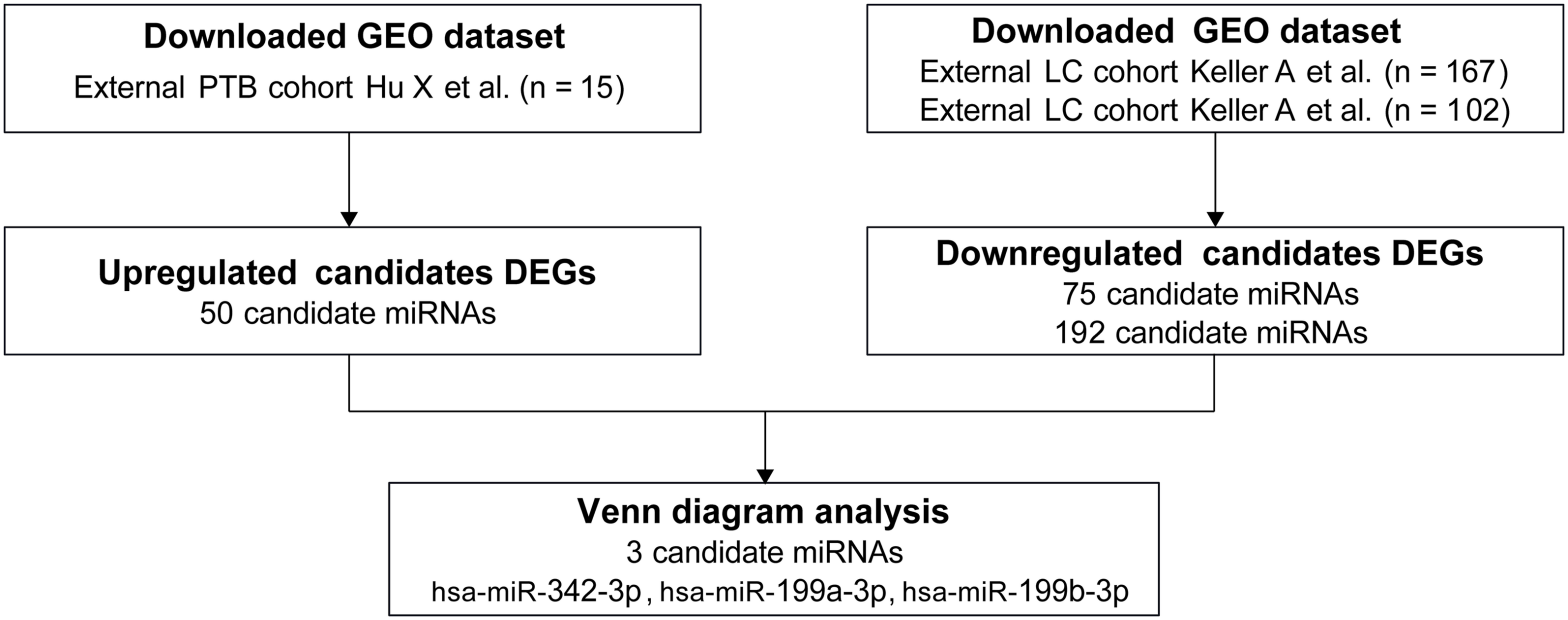
miRNA panel selection. PTB, pulmonary tuberculosis; LC, lung cancer.

The primary objective of this study was to identify a robust miRNA signature capable of distinguishing PTB from LC. In the current study, GEO datasets were analyzed, and 50 differentially expressed miRNAs were identified in a PTB dataset, along with 75 and 192 in two independent LC datasets. Comparative analysis revealed six overlapping candidates: hsa‑miR‑199a‑3p, hsa‑miR‑199b‑3p, hsa‑miR‑342‑3p, hsa‑miR‑502‑3p, hsa‑miR‑361‑5p, and hsa‑miR‑423‑5p. Because hsa-miR‑502‑3p, hsa‑miR‑361‑5p, and hsa‑miR‑423‑5p were consistently upregulated in both PTB and LC, they lacked discriminatory specificity and were excluded. Therefore, hsa‑miR‑342‑3p, hsa‑miR‑199a‑3p, and hsa‑miR‑199b‑3p were selected as the candidate biomarker panel (Figure 2).

**Figure 3:**
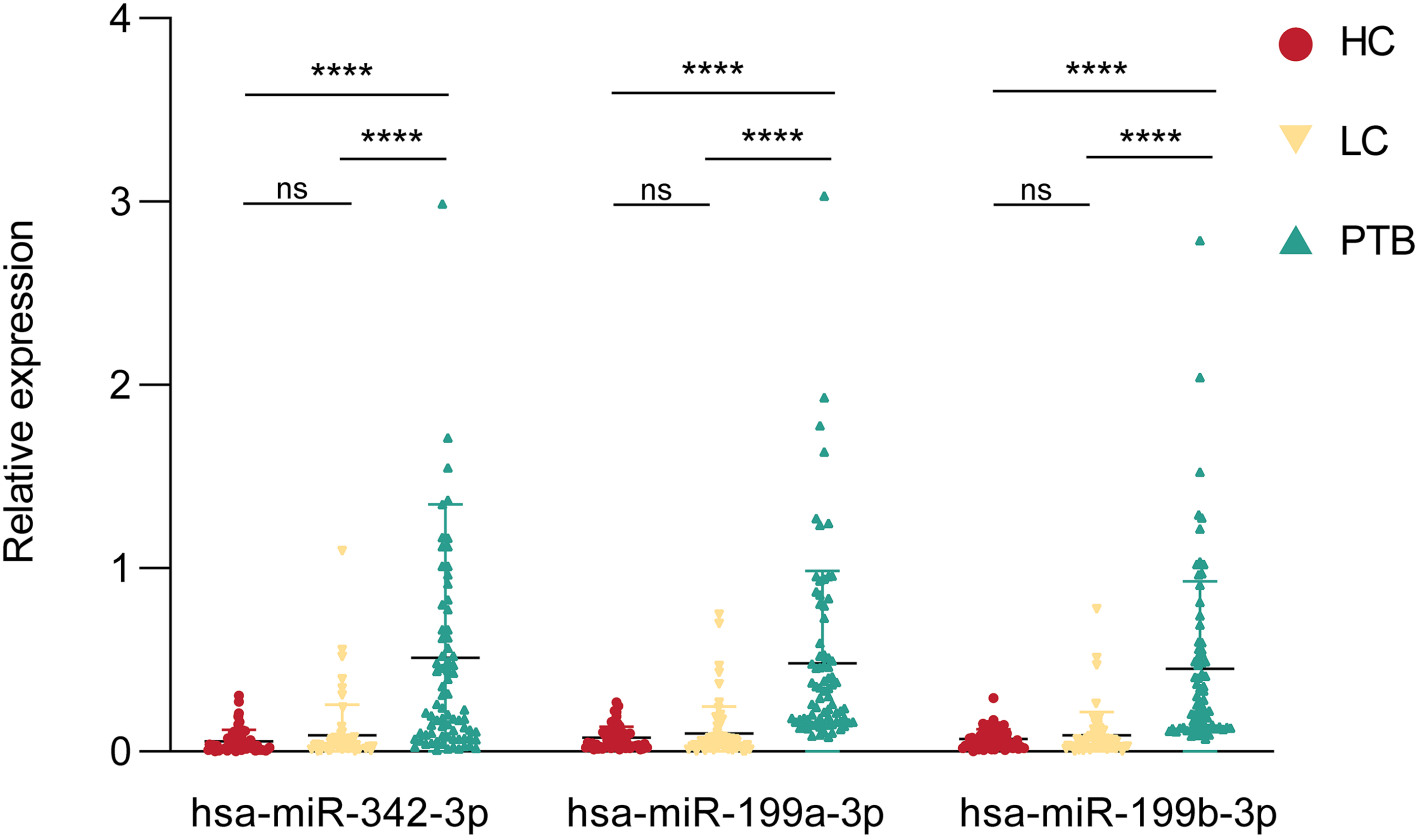
Profiling of blood miRNA expression in the clinical cohort. The clinical cohort consisted of a training group (PTB, *n* = 77; LC, *n* = 68) and an HC group (*n* = 65). PTB, pulmonary tuberculosis; LC, lung cancer; ns, no significance; **** *p* < 0.0001.

To establish a rapid, non‑invasive diagnostic model, the plasma expression levels of these three miRNAs were quantified by RT‑qPCR. Significant differences were observed in expression levels between PTB patients (n = 77) and LC patients (n = 68), as well as between PTB patients and healthy controls (HC, n = 65) (Figure 3), supporting their potential clinical applicability.

### Establishment and validation of a diagnostic model of the 3-miRNA panel

The diagnostic model was trained on the cohort using binary logistic regression analysis [n = 145 (77 PTB and 68 LC)]. The diagnostic score for each case was calculated using the following equation:


$$\begin{array}{l} \text{miRNA risk score}=\\ \frac{1}{1+e^{-(-1.646+0.462\cdot \mathrm{miR}-342-3p+4.193\cdot \mathrm{miR}-199a-3p+5.023\cdot \mathrm{miR}-199b-3p)}} \end{array}$$

**Figure 4:**
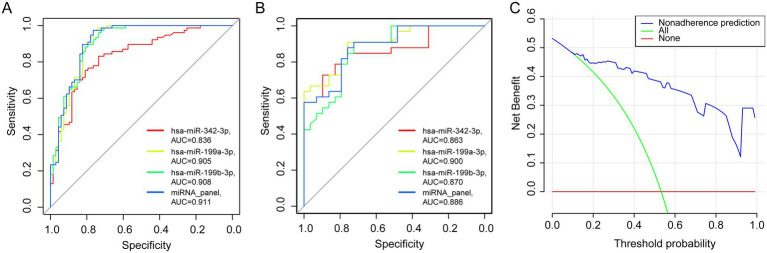
Training and validation of the miRNA panel. **(A,B)** The area under the ROC curve of the training (PTB, *n* = 77, LC, *n* = 68) and validation cohorts (PTB, *n* = 33, LC, *n* = 29). **(C)** Decision curve analysis for evaluating the performance of the 3-miRNA panel.

The diagnostic model demonstrated excellent performance in plasma samples from patients with PTB, yielding an AUC of 0.911 (95% CI: 0.852–0.952, *p* < 0.001; Figure 4A), with a sensitivity of 0.974 and a specificity of 0.765. Based on these findings from the training cohort, the model was further evaluated for robustness and accuracy in an independent validation cohort (*n* = 62; 33 patients with PTB and 29 patients with LC). Validation testing confirmed the strong diagnostic application of the model, with an AUC of 0.886 (95% CI: 0.780–0.953, *p* < 0.001), a sensitivity of 0.879, and a specificity of 0.759 (Figure 4B).

For comparison, models constructed using only two miRNAs were also assessed; however, their AUC values were lower than those of the three-miRNA model, as summarized in Supplementary Table 1.

### MiRNA panel: significant benefit in clinical practice

The clinical utility of any diagnostic tool depends on its ability to maximize accurate detection while minimizing the risks associated with misdiagnosis. In suspected LC cases, invasive procedures are often necessary, and both false-positive and false-negative results can lead to severe clinical consequences. To assess the practical applicability of the three-miRNA panel, DCA was performed (Figure 4C). In the test cohort, the DCA demonstrated that the miRNA panel consistently provided a higher net clinical benefit across all threshold probabilities compared with the default strategies of treating all patients or treating none. These results suggest that the panel may offer tangible clinical advantages by reducing the need for unnecessary invasive procedures and lowering the risk of diagnostic errors.

### Age-related impact on miRNA expression and diagnostic performance

**Figure 5:**
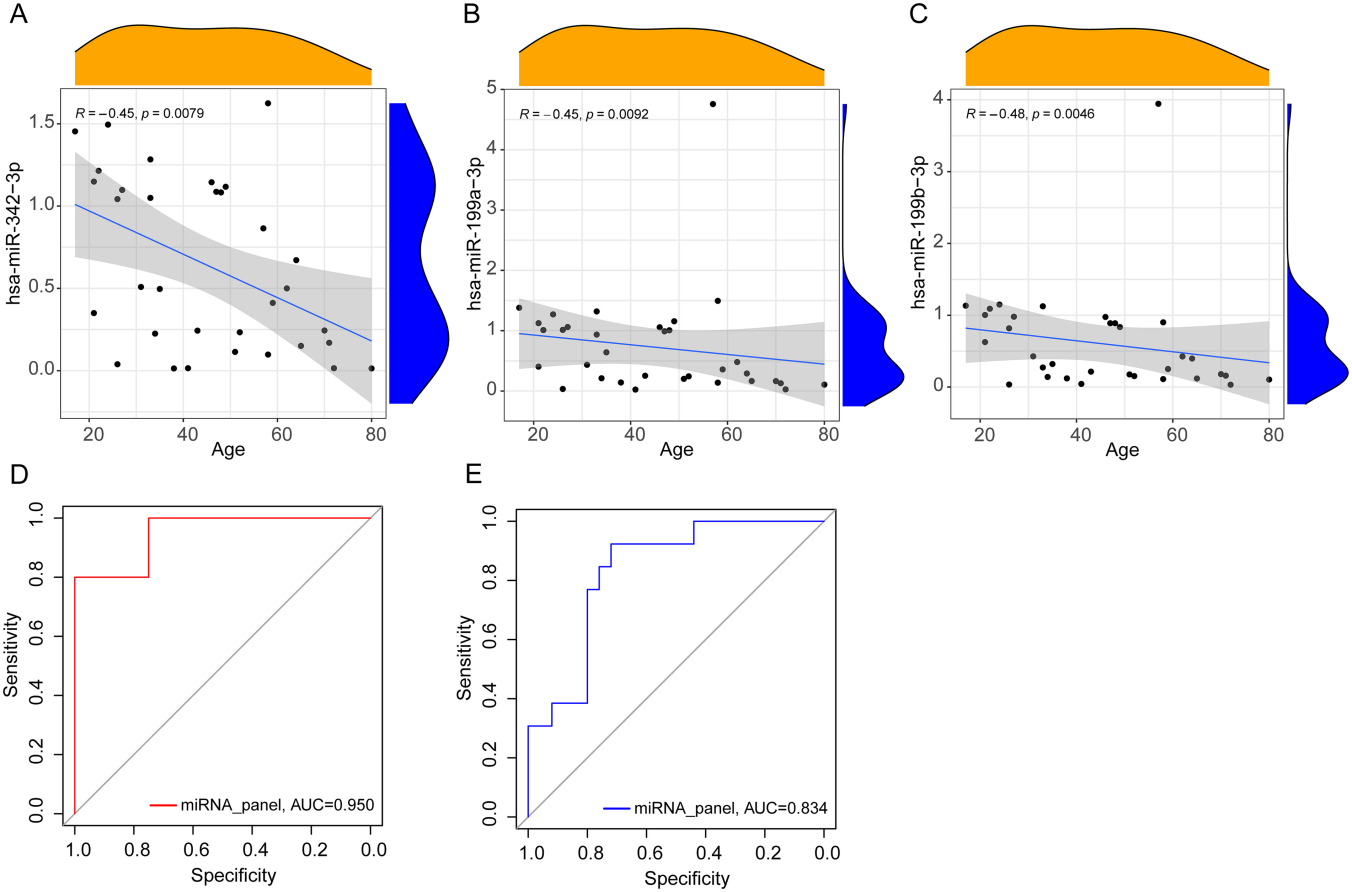
Correlation analysis and ROC curves. **(A–C)** Spearman correlation analysis between miRNA relative expression levels and age (PTB, *n* = 33). **(D,E)** The area under the ROC curve of the younger (PTB, *n* = 20, LC, *n* = 4) and older group (PTB, *n* = 13, LC, *n* = 25).

To examine the potential influence of age on miRNA expression and its implications for diagnostic accuracy, correlations between age and the expression levels of the three selected miRNAs were analyzed in patients with PTB. A negative correlation was observed, with expression levels decreasing as age increased (Figures 5A–C). The correlation coefficients were R = −0.45 (*p* = 0.008) for hsa-miR-342-3p, R = −0.45 (*p* = 0.009) for hsa-miR-199a-3p, and R = −0.48 (*p* = 0.005) for hsa-miR-199b-3p.

To further assess the effect of age on diagnostic performance, participants were stratified into two groups: younger (≤ 50 years) and older (≥ 51 years). The ROC curve analysis indicated improved diagnostic performance in the younger group, with an AUC of 0.950, compared with an AUC of 0.834 in the older group (Figures 5D,E). These results suggest that the diagnostic application of the miRNA panel may be particularly enhanced in younger individuals.

### High sensitivity and early recognition in PTB compared to conventional methods

Early detection of PTB is critical for improving clinical outcomes. In the validation cohort, patients were initially stratified into early or late stages according to the presence or absence of pulmonary cavities. Recognizing that this single parameter has inherent limitations and may not adequately reflect the overall disease status, additional severity-related clinical indicators, including monocyte count, hemoglobin (Hb) levels, C-reactive protein (CRP), and erythrocyte sedimentation rate (ESR), were also evaluated to provide a more comprehensive assessment (20–24).

**Figure 6:**
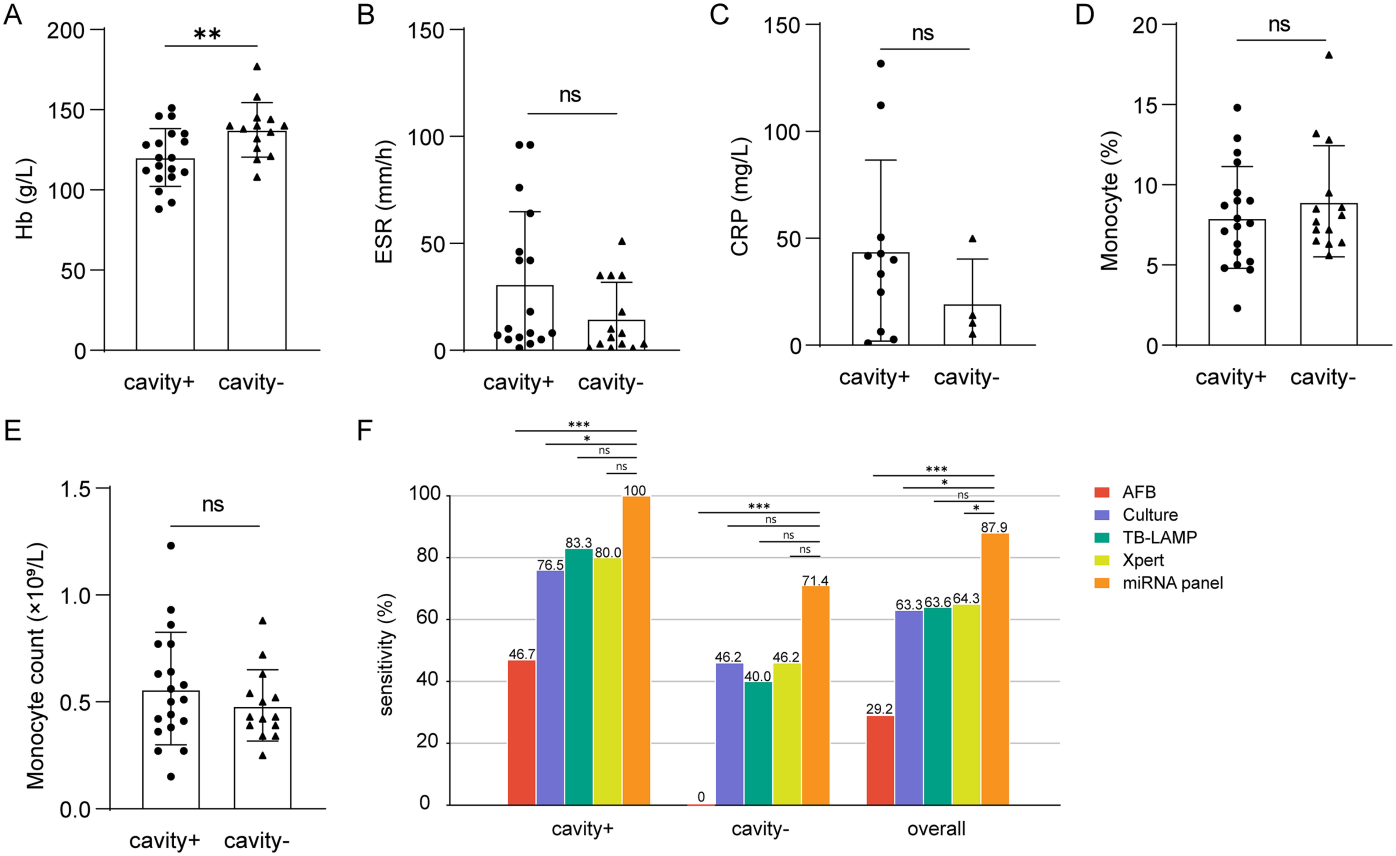
Differences in severity-related indicators and sensitivity comparison of different groups. **(A–E)** Hb (cavity+, *n* = 19, cavity−, *n* = 14), ESR (cavity+, *n* = 17, cavity-, *n* = 14), CRP (cavity+, *n* = 11, cavity−, *n* = 4), monocyte percentage (cavity+, *n* = 19, cavity−, *n* = 14) and count (cavity+, *n* = 19, cavity−, *n* = 14). **(F)** Comparison of miRNA panel, AFB smear, MTB culture, TB-LAMP, and Xpert assays among patients with or without cavity. ns, no significance. * *p* < 0.05. ** *p* < 0.01. *** *p* < 0.001.

In PTB patients with cavitary disease, ESR, CRP, and monocyte counts were elevated compared with non-cavitary cases, whereas hemoglobin levels were significantly reduced (Figures 6A–E). These results underscore apparent clinical differences between cavitary and non-cavitary stages of PTB. The diagnostic utility of the three-miRNA panel was then compared with conventional methods. Across PTB subgroups, the panel consistently demonstrated high sensitivity. In cavitary PTB, it significantly outperformed smear microscopy and culture in detection sensitivity (*p* < 0.05). For non-cavitary PTB, the panel achieved sensitivity comparable to culture, although it did not surpass Xpert MTB/RIF (*p* < 0.05). When all PTB cases were assessed together, the miRNA panel showed higher sensitivity than Xpert MTB/RIF, smear microscopy, and culture (*p* < 0.05). These findings suggest that the panel may serve as a viable diagnostic alternative in clinical practice, with performance matching or, in some cases, exceeding that of Xpert (Figure 6F).

### Functional and pathway analysis of hsa-miR-342-3p and hsa-miR-199a/b-3p

To investigate the potential biological roles of the selected miRNAs in TB, GO enrichment analysis was conducted on their predicted target mRNAs. Target genes were identified based on the intersecting results from three prediction databases: TargetScan 7.2, miRWalk, and miRDB.

**Figure 7:**
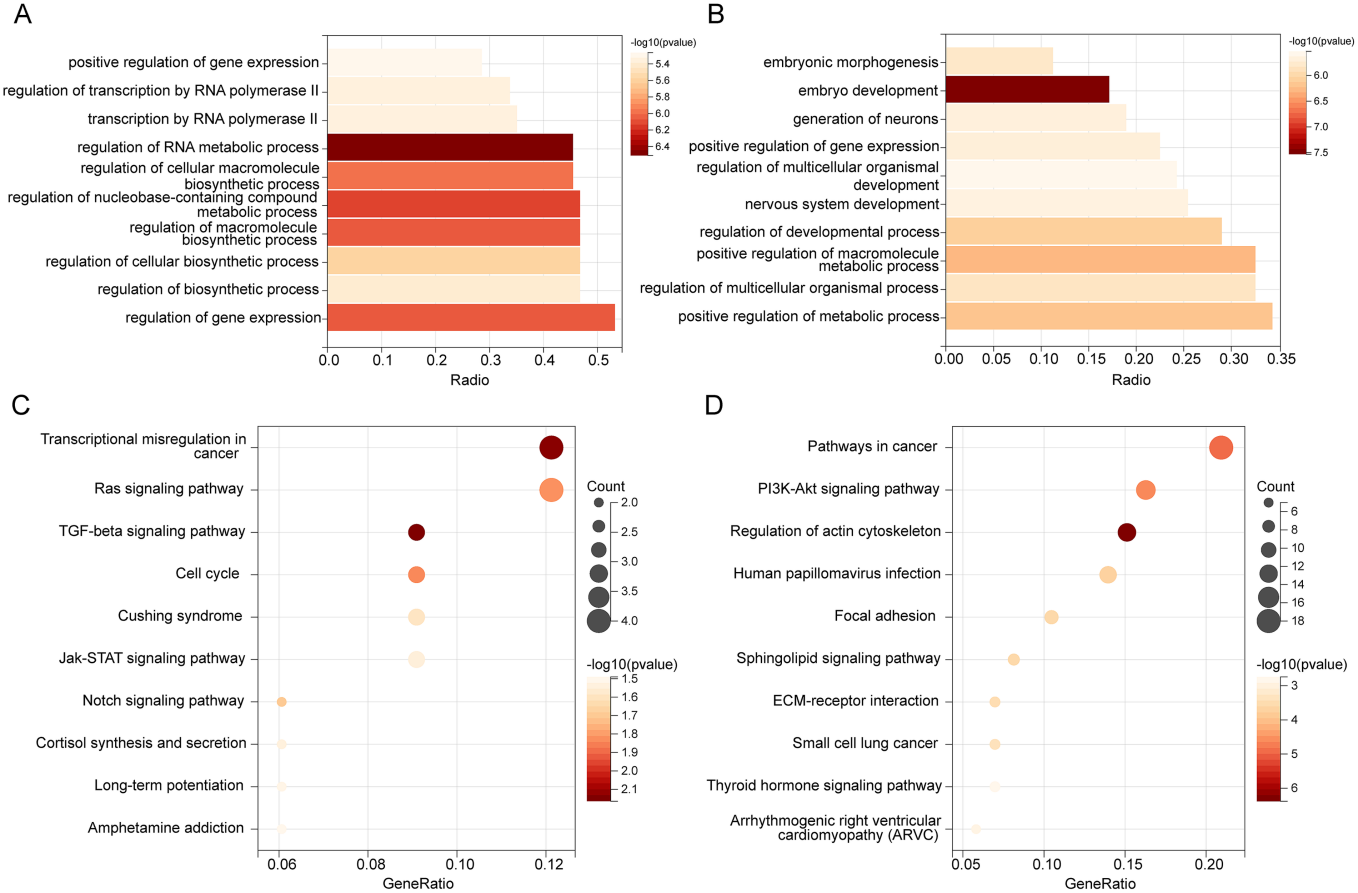
GO and KEGG enrichment. Bar plot shows the biological process (BP) of GO Enrichment Analysis with target mRNAs of hsa-miR-342-3p **(A)** and hsa-miR-199a/b-3p **(B)**. **(C)** KEGG pathway enrichment of hsa-miR-342-3p. **(D)** KEGG enrichment of hsa-miR-199a/b-3p.

The GO analysis revealed that target genes of hsa-miR-342-3p were significantly enriched in biological processes, including positive regulation of gene expression, transcriptional regulation by RNA polymerase II, and RNA metabolic regulation (Figure 7A). Similarly, target genes of hsa-miR-199a/b-3p were associated with pathways involved in positive regulation of gene expression, macromolecule metabolic processes, and overall metabolic regulation (Figure 7B).

Then, KEGG pathway enrichment analysis was performed to identify signaling pathways that these miRNAs may regulate. Genes targeted by hsa-miR-342-3p were mainly enriched in the Ras, TGF-*β*, and Jak–STAT signaling pathways (Figure 7C). However, target genes of hsa-miR-199a/b-3p showed significant enrichment in the PI3K-Akt, thyroid hormone, and sphingolipid signaling pathways (Figure 7D).

## Discussion

In clinical practice, distinguishing PTB from LC in patients with overlapping radiological presentations remains a significant challenge, as conventional non-invasive diagnostic methods are often limited by prolonged turnaround times and insufficient sensitivity. An effective diagnostic tool should ideally be rapid, affordable, and non-invasive, enabling the timely and accurate differentiation of conditions. Addressing the limitations of current strategies necessitates the development of innovative approaches that improve diagnostic precision. Although recent efforts in non-invasive rapid detection have primarily concentrated on advancements in radiological techniques (25), their diagnostic accuracy remains inadequate and requires further refinement.

In this context, molecular biomarker-based detection strategies are gaining increasing attention as a promising alternative. Extensive evidence suggests that miRNAs play a crucial role as key regulators in both infectious immune responses and tumor progression. The biological functions of the miRNA panel identified in this study are closely aligned with these pathological processes. For instance, the predicted target genes of hsa-miR-342-3p are highly enriched in pathways regulating gene expression, particularly the TGF-*β* and JAK–STAT signaling cascades, which is consistent with its established roles in tuberculosis and cancer biology. In PTB, hsa-miR-342-3p enhances host defense by targeting SOCS6, a negative regulator of the JAK–STAT pathway, promoting the secretion of pro-inflammatory cytokines and inducing macrophage apoptosis (16, 26). It’s observed that upregulation in PTB may therefore represent a host-protective response. By contrast, in NSCLC and other solid tumors, hsa-miR-342-3p functions as a tumor suppressor, inhibiting oncogenic drivers such as AGR2 and FOXQ1, while its association with the TGF-β pathway suggests a role in modulating the tumor microenvironment (27, 28). The frequent downregulation of hsa-miR-342-3p in cancer is likely attributable to the immunosuppressive conditions of the tumor microenvironment, which attenuate its tumor-suppressive activity.

Members of the hsa-miR-199 family (hsa-miR-199a-3p and hsa-miR-199b-3p) display enrichment in metabolic regulation and cancer-associated pathways. In models of septic acute kidney injury, these miRNAs modulate cellular homeostasis by suppressing Nrf2-mediated antioxidant gene expression (29, 30). Given the elevated oxidative stress associated with TB infection, this mechanism may contribute to the pathophysiology of PTB. The upregulation of hsa-miR-199a/b-3p in PTB serum likely reflects a compensatory host response aimed at curbing excessive inflammation while maintaining Nrf2-dependent antioxidant defenses. In NSCLC, the hsa-miR-199 family acts as a tumor suppressor primarily by inhibiting the Rheb-mTOR axis, a central pathway governing metabolic homeostasis and biosynthesis (31). Its downregulation in cancer may similarly result from the immunosuppressive tumor microenvironment, which impairs tumor-suppressive signaling.

The miRNA panel assessed in this study demonstrated robust diagnostic accuracy for distinguishing PTB from LC across both training and validation cohorts, with sensitivity and specificity comparable to, and in some instances superior to, those of several commonly used biomarkers. In comparison with cell-free DNA (cfDNA)-based detection methods, the panel showed higher sensitivity, largely because cfDNA released from tuberculous necrotic foci often overlaps with tumor-derived cfDNA, reducing the discriminative value of quantitative cfDNA analysis (32). Regarding circular RNAs (circRNAs), their application in differential diagnosis remains mainly exploratory. Currently, no reliable circRNA markers with stable and consistently opposite expression patterns between PTB and LC have been identified. Furthermore, the complexity and high cost of circRNA detection further limit their clinical applicability at the primary care level (33). Furthermore, when compared with combined tumor marker detection, miRNA expression offers distinct advantages, as it is less influenced by inflammation and demonstrates greater stability and reproducibility in clinical samples (34). These attributes underscore the superior overall performance and translational potential of the identified miRNA panel.

Beyond these comparisons, the panel’s sensitivity for early PTB detection was further evaluated against conventional diagnostic methods, including culture, Xpert assay, AFB smear microscopy, and LAMP, in clinical settings. For this purpose, patients without pulmonary cavities were classified as having early-stage PTB, while those with cavities were considered to have late-stage PTB. Across both stages, the panel demonstrated diagnostic performance comparable to, and in some cases exceeding, that of Xpert, particularly in early disease detection, where reliable differentiation is most critical.

Although Xpert remains a widely validated diagnostic platform, our results suggest that the proposed approach could serve as a valuable complementary tool, particularly in resource-constrained settings or in scenarios where rapid diagnosis is critical. Numerous studies have confirmed the remarkable stability of miRNAs under diverse storage conditions: they can remain preserved for more than 17 years at −80 °C, remain stable for up to 7 days at 4 °C, and exhibit no significant degradation for at least 24 h at room temperature. These characteristics fulfill the stringent criteria required for reliable clinical diagnostics (35, 36). In the present study, all plasma samples were either processed for immediate miRNA extraction or subjected to a single freeze–thaw cycle at −80 °C within 1 hour of collection. Further, stringent quality control procedures were implemented throughout all pre-analytical stages to minimize technical variation, ensuring both analytical precision and reproducibility.

However, several limitations of this study should be acknowledged. First, patients with concurrent PTB and LC were excluded due to the rarity of such cases; as a result, miRNA expression patterns in this subgroup were not evaluated. Second, since LC patients do not typically undergo TB-specific diagnostic testing, direct comparisons of TB detection specificity across different diagnostic methods could not be performed. Moreover, although efforts were made to minimize confounding factors during sample selection, such as excluding individuals with co-infections, other variables, including concurrent infections and age-related differences, may still influence the diagnostic accuracy of the miRNA panel in real-world clinical settings.

To address these gaps, future studies should expand cohort sizes and incorporate more diverse patient populations, while also benchmarking the panel against emerging state-of-the-art diagnostic technologies. Such investigations will be essential to further validate and refine the clinical translational potential of this miRNA-based diagnostic strategy.

## Conclusion

In summary, this study established a rapid and non-invasive diagnostic strategy capable of differentiating PTB from LC in patients with overlapping radiological presentations. The identified miRNA panel demonstrated higher sensitivity compared to conventional methods, including Xpert MTB/RIF, smear microscopy, and culture, while also offering a lower cost and greater feasibility. These attributes highlight its strong potential for clinical translation, particularly in resource-limited settings, where it may serve as a practical and effective tool for diagnosing focal pulmonary lesions.

## Data Availability

The raw data supporting the conclusions of this article will be made available by the authors, without undue reservation.

## Footnotes

^1^ https://www.ncbi.nlm.nih.gov/geo/

^2^ https://www.ncbi.nlm.nih.gov/geo/geo2r/

^3^ https://www.targetscan.org/vert_72/

^4^ http://mirwalk.umm.uni-heidelberg.de/

^5^ http://www.mirdb.org/

^6^ http://sangerbox.com/
