# Supplementary material for: A novel circulating miRNA panel for early differential diagnosis of pulmonary tuberculosis from lung cancer with similar radiographic presentations
Source: Front Med (Lausanne). 2025 Oct 8;12:1660291. doi: 10.3389/fmed.2025.1660291 (PMC12540171; doi:10.3389/fmed.2025.1660291)
Supplement: Supplementary file 1 [file Table_1.docx]

**Supplementary Table 1.** AUC Values for Diagnostic Models of Two-Molecule Combinations

| miRNA panel | AUC | 95% CI |
| --- | --- | --- |
| miR-342-3p+miR-199a-3p | 0.909 | 0.850 - 0.951 |
| miR-342-3p+miR-199b-3p | 0.909 | 0.849 - 0.950 |
| miR-199a-3p+miR-199b-3p | 0.908 | 0.849 - 0.950 |
